# Risk of Secondary Household Transmission of COVID-19 from Health Care Workers in a Hospital in Spain

**DOI:** 10.3390/epidemiologia3010001

**Published:** 2021-12-27

**Authors:** Miren Remón-Berrade, Sara Guillen-Aguinaga, Isabel Sarrate-Adot, Maria Pilar Garcia-Garcia, Maria del Carmen Lerga-Berruezo, Laura Guillen-Aguinaga, Francisco Guillen-Grima

**Affiliations:** 1 Department of Preventive Medicine, Clínica Universidad de Navarra, 31008 Pamplona, Navarra, Spain; mremon@unav.es (M.R.-B.); misarrate@unav.es (I.S.-A.); mpgar@unav.es (M.P.G.-G.); mlergab@unav.edu (M.d.C.L.-B.); 2 Azpilagaña Health Center, Navarra Health Service, 31006 Pamplona, Navarra, Spain; sguillen.4@alumni.unav.es; 3 Department of Health Sciences, Public University of Navarra (UPNA), 31008 Pamplona, Navarra, Spain; 4 Department of Nursing, Clínica Universidad de Navarra, 31008 Pamplona, Navarra, Spain; lguillen@unav.es; 5 Instituto de Investigación Sanitaria de Navarra (IdiSNA), 31008 Pamplona, Navarra, Spain

**Keywords:** COVID-19, secondary attack rate, risk-factors, household contacts, health care workers

## Abstract

Background: Hospital health care workers are at high risk of developing COVID-19 and transmitting the disease to their family upon returning home; the aim here is to estimate the secondary attack rate of COVID-19 in household contacts of health care workers and their transmission risk factors. Material and Methods: COVID-19 cases in the health care workers of an academic hospital in Pamplona, Spain, from 2 March to 26 May 2020, were followed up. The secondary attack rate (SAR) was estimated from cases in household contacts of index cases and their risk factors by Poisson regression. Results: 89 index cases were studied from 99 notified cases in health care workers (89.0%), excluding secondary cases or those who lived alone. Forty-six secondary cases confirmed by the laboratory were found among 326 household contacts, a secondary attack rate of 14.11% (95% CI 10.75–18.31), and 33 household contacts with acute infection symptoms without microbiologic confirmation 10.12% (95% CI 7.30–13.87). Considering all the cases, the secondary attack rate was 27.3 (95% CI 22.75–32.38). Risk factors were the gender and profession of the index case, the number of people living in the household, and the number of persons per bedroom. When the index case health worker used a single room, it had a protective effect, with an incidence rate ratio (IRR) of 0.493 (95% CI 0.246–0.985); Conclusions: The secondary attack rate found among household contacts of health care workers is high. The preventive isolation of health care workers in individual rooms in their house may reduce the transmission in their families.

## 1. Introduction

COVID-19 is a disease caused by SARS-CoV-2. It was first reported in Wuhan, China, in December 2019 and then spread across continents. In Spain, the first case was on 31 January, from a German tourist on the Canary Island of La Gomera, and the second on 9 February, from a British tourist in Palma de Mallorca, Balearic Islands. The incubation is between 2 and 14 days. Some patients may be asymptomatic, some may develop mild symptoms, and some may develop severe pneumonia [1].

SARS-CoV-2 is highly contagious, and numerous studies have been conducted to learn the secondary attack rate in families in which a member develops COVID-19 [2,3]. According to WHO, health care workers (HCW) are defined as “all persons who perform actions whose primary intention is to improve health” [4,5]. Health care workers include physicians, nurses, nursing assistants, orderlies, support staff (cleaning, laundry, kitchen, maintenance, and security), and hospital managers [4]. Health care workers are at a high risk of developing COVID-19 and may even die from their occupation [6,7]. Numerous studies have been conducted on the risk of infection in healthcare workers. Nearly 25% of health care workers said they were reluctant to work during a pandemic because of the risk of transmitting the disease to their families [8]. Health care workers have experimented with anxiety, high levels of psychological distress, and mental disorders, such as depression, generalized anxiety disorder (GAD), and posttraumatic stress disorder (PTSD), during the pandemic [9,10]. This can lead to burnout in health care workers [11]. The stress level is higher in nurses than in other health care workers [12]. One of the causes was concern about transmitting the disease to their family upon returning home [13], especially when they have vulnerable family members [14]. Despite the importance of this fact, few publications deal with the risk of transmission from healthcare workers to their families [15,16,17].

Objective: This study aims to estimate in an academic hospital in Navarra (Spain) the secondary attack rate of COVID-19 in household contacts of health care workers and their transmission risk factors.

## 2. Materials and Methods

As part of the epidemiological surveillance of COVID-19, the Department of Preventive Medicine of the Clinica Universidad de Navarra in Pamplona studied the cases of COVID-19 among health care workers. A retrospective cohort was also made with family contacts of health care workers.

When a case was reported in health care workers, an epidemiological survey was completed. Follow-up of patients and their contacts were performed by contact tracers using the COVID-19 protocols approved by the Ministry of Health [18]. These protocols involved the clinical investigation of close contacts and their quarantine. In the first 24 h, PCR or antigen testing was performed if reagents were available. Between 2 March and 26 May 2020, during the first wave of the pandemic of COVID-19 in Pamplona, ninety-nine cases of COVID-19 were diagnosed among health care workers at Clinica Universidad de Navarra. We computed the primary attack rate among health care workers at Clinica Universidad de Navarra. Health care workers were divided into five groups: physicians, nurses, nursing assistants, orderlies, and others.

All subjects provided oral informed consent to the processing of the telephone interview data for research purposes. The research was conducted in compliance with the principles of the Helsinki Declaration as part of the mandatory epidemiological surveillance of COVID-19 among health care workers. A telephone survey was used to follow up on the evolution of family contacts. We collected demographic information, housing conditions, the number of people living in the dwelling, and whether the health care workers had taken extraordinary preventive measures before being diagnosed. 

Primary attack rates (AR) among health care workers (HCW) and secondary attack rates (SAR) among the household contacts of affected workers have been calculated.

AR (%) = Number of new cases of COVID-19 in HCW/Total number of HCW at risk × 100

SAR (%) = Number of new cases among contacts/Total number of contacts at risk × 100

Odds ratios and 95% confidence intervals were calculated with IBM SPSS version 22. Poisson regression was performed to relate the different exposures to the number of secondary cases by calculating the univariate incidence rate ratio with Stata version 13.

## 3. Results

**Figure 1:**
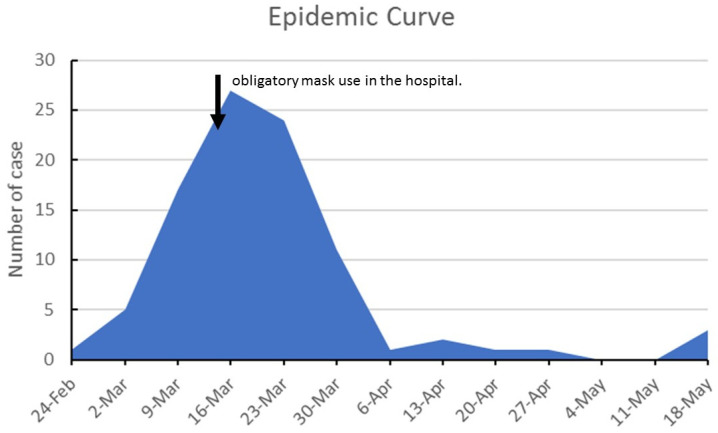
Epidemic Curve of cases in Health Care workers February–May 2020.

The maximum number of cases among health care workers occurred in the week of 16 March (Figure 1). On 12 March 2020, the hospital management imposed all staff’s mandatory face masks. After that, there was an abrupt decrease in the number of cases.

The primary attack rate among health care workers was 4.51% (95% CI 3.72–5.47). The most affected groups were hospital orderlies, with an AR of 15.38% and an OR = 18.73% (95% CI = 3.63–96.73), followed by nurses and nursing assistants with attack rates of 7.15% and 6.62%. The least affected group was the cleaning, laundry, and kitchen staff, with an AR of 0.96%. 

A total of 99 workers had COVID-19. Five workers could not be reached after five successive telephone calls (they were on holiday or hospitalized with COVID-19). Among the remaining 94 health care workers, five were excluded as index cases to estimate secondary attack rates (SAR) because they lived alone or were secondary cases.

In the secondary attack rate study, 89 index cases were included, representing 89.9% of the notified cases. Forty-six secondary patients confirmed by the laboratory were found among 326 household contacts. This means a secondary attack rate of 14.11% (95% CI 10.75–18.31). In addition to the laboratory-confirmed cases, 33 household contacts with symptoms classified as secondary symptoms only made up 10.12% (95% CI 7.30–13.87). Considering all the cases, the secondary attack rate was 27.3 (95% CI 22.75–32.38).

**Figure 2:**
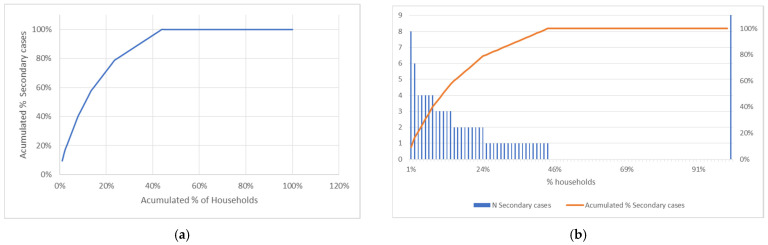
Distribution of cases by households: (**a**) Secondary cases by households (**b**) Pareto’s chart of secondary cases of COVID-19 by households.

Secondary cases are concentrated in some families. Thus, in 11% of the households, more than 50% of the cases of secondary transmission are concentrated. (Table S1). In the same way, 25% of the families account for 80% of the cases of secondary transmission (Figure 2). 

### 3.1. Index Cases Characteristic and Risk of Secondary Transmission

The univariate Poisson regression shows that secondary transmission was more frequent in males and the 30–39 age group with incidence rate ratios of 1.351 and 2.054. The professional groups with the highest risk of having secondary infections at home were physicians with an IRR of 5.777 and orderlies with an IRR of 4.800 (Table 1 and Table 2).

### 3.2. Household and Risk of Secondary Transmission

In a univariate Poisson regression model, households with four or more members have a higher risk of having secondary cases. The highest risk appears in those households with more than five people with an incidence rate ratio of 7.064 (Table 3). The number of bedrooms and bathrooms in the house is not associated with having secondary cases. When the number of persons per bedroom was higher than 1.5, the incidence rate ratio of having COVID-19 cases was 5.167. The number of persons per bathroom has a lower influence on secondary transmission.

In Table 4, we present the hygienic measures taken by health care workers at home. The IRR of having secondary cases of COVID-19 was lower when using masks at home and gloves (IRR = 0.607; 95% CI 0.362–0.988), cleaning surfaces with disinfectant, and having individual bedrooms. Using individual or disposable tableware, having a separate bathroom, and washing clothes separately were not associated with having secondary cases of COVID-19 in the household.

In the multivariate Poisson model, we adjusted by age, sex, profession, and onset date. We computed Poisson regression models only with those variables that were significant in the univariate model. Wearing a mask and gloves at home, using individual or disposable tableware, cleaning surfaces with disinfectant, and having a single room all contribute to reducing the risk of secondary transmission (Table 5). 

## 4. Discussion

A meta-analysis found that the prevalence of COVID-19 among health care workers was 10.1% but was higher in the United States with 17.1% [19]. A study found that 19.4% of health care workers in New York were infected with COVID-19. In a hospital in Switzerland, the seroprevalence among health care workers was 10.0% [20,21]. Nurses are one of the most affected groups among healthcare workers. A meta-analysis found that the prevalence of anti-COVID-19 antibodies was 10.3% among nurses in Europe [22]. In our study, the risk was higher in nurses than in physicians. This differs from another meta-analysis that found that physicians were more at risk than nurses [23]. Our primary attack rate, 7.79%, was lower than the 9.5% of a Swiss hospital [21]. It was higher than that of the meta-analysis mentioned above, 6.5% [23]. In our study, the group with a higher attack rate was orderly with 18.73%, while in the Swiss study, the group with a higher prevalence was trainees/students [21].

The reference group was the cleaning, kitchen, and laundry staff, most of whom were not in contact with patients. Although some of them did not, the cleaning staff in the ICU and on the floor with COVID-19 patients could contact COVID-19 patient rooms. However, this personnel used all the personal protective equipment and available ultraviolet disinfection systems. 

Similarly, the “other professionals” group included administrative, managerial, maintenance (electricians, computer technicians, plumbers, carpenters), radiology technicians, laboratory technicians, and CAT technicians. This group could also have been considered baseline because most of them were not in contact with patients, although they might have some contact with patients on occasion, such as making a repair in a room or taking a chest X-ray of a patient with COVID-19.

Table 1 shows that the attack rate of the other group is very similar to that of the cleaning, kitchen, and laundry group. It would have been possible to merge the two groups, but we felt it was more informative to present them separately.

There is evidence that COVID-19 transmission and secondary transmission occur in clusters [24,25,26], sometimes due to super-spreading events [25]. We have found that a small proportion of health care workers was responsible for most of the secondary transmission.

Transmission is frequent in households in the general population. One meta-analysis found that families have the highest transmission rates, with a pooled secondary attack rate of 21.1% [3]. In Spain, the secondary attack rate in households was 38.7% [27]. 

The rate found among the families of healthcare personnel in our study is like that of the meta-analysis in the general population but substantially lower than the Spanish figures, indicating that the preventive measures taken by healthcare professionals have been partially effective. We detected a secondary attack rate of 27.3% in our study. In Spain, a survey among health care workers’ children found a secondary attack rate of 43.7% [28]. In comparison, studies performed among health care workers’ families in Amsterdam and China detected secondary attack rates of 10.98%, 18.8%, and 23.3% [29,30,31]. 

Table 3 shows in the univariate Poisson regression that the number of cases is related to the number of people living in the house and the number of bedrooms and bathrooms. The number of bedrooms and bathrooms in the dwelling is related to the number of people. The observations must be independent in Poisson regression, which is not the case here. Table 3 shows that the higher the number of people in a household, the higher the number of cases predicted by the model. This result shows the nonindependence of the cases, and so one should be careful when interpreting Table 2 and Table 4. It is necessary to protect both the health care workers and their families [32]. Because of the possibility of infection during the incubation period, the measures to be effective must be permanent and heavy [8]. Our study, despite its limitations, sheds light on the most effective measures: wearing a mask and gloves at home, using individual or disposable tableware, cleaning surfaces with disinfectant, and having a single room. 

During the pandemic, some hotels provided housing for health care workers [33,34,35,36,37,38,39]. In one survey, 77% of health care workers stated that they could not isolate themselves in their household [40]. Our findings suggest that this measure may be helpful in future pandemic waves to protect the families of health care workers who do not have the space in their homes to have an individual room for the HCW.

## Figures and Tables

**Table 1:** Attack rate in health care workers.

Profession	*n*	AR	OR	95% CI
Cleaning, laundry, and Kitchen staff	208	0.96%	1	
Physicians	475	4.00%	4.29	0.99–18.60
Nurses	657	7.15%	7.94	1.91–32.96
Nursing assistant	298	6.04%	6.62	1.52–28.85
Orderlies	39	15.38%	18.73	3.63–96.73
Other professionals	495	1.21%	1.26	0.25–6.31

**Table 2:** Univariate Exact Poisson regression of secondary transmission by personal characteristics of index cases.

Variable	Case	No Case	Total	IRR	95% CI	*p*
Age				0.993	0.975–1.012	0.492
Age Group						
18–29	19	31	50	1.180	0.591–2.353	0.638
30–39	23	23	46	2.054	1.056–3.990	0.034
40–49	29	126	155	1.380	0.729–2.613	0.321
50–62	14	61	75	1		
Sex						
Female	10	79	89	1		
Male	75	162	237	1.351	1.060–1.725	0.015
Occupation						
other professionals	2	39	41	1	-	-
Physician	26	89	115	5.777	1.371–24.342	0.017
nurse	36	82	118	3.512	0.845–14.586	0.084
auxiliary	15	29	44	3.529	0.807–15.433	0.094
orderlies	6	2	8	4.800	0.968–23.781	0.055

**Table 3:** Univariate Exact Poisson regression of secondary transmission by household characteristics of index cases.

Variable	Case	No Case	Total	IRR	95% CI	*p*
*n*. Household members						
2	6	13	19	1		
3	9	27	36	1.583	0.563–4.448	0.383
4	21	51	72	2.770	1.118–6.865	0.028
5	20	40	60	1.965	1.696–10.513	0.002
>5	29	110	139	7.064	2.932–17.014	<0.001
*n*. Bedrooms						
2	8	13	21	1		
3	32	53	85	2.133	0.983–4.629	0.055
4	22	57	79	1.760	0.783–3.953	0.171
>4	15	113	128	2.000	0.847–4.717	0.113
*n*. Bathrooms				0.973	0.902–1.050	0.486
*n*. Persons/Bedroom						
<1	8	13	21	1		
1	32	53	85	2.213	0.908–5.396	0.080
1–1.499	22	57	79	2.125	0.825–5.477	0.119
≥1.5	15	113	128	5.167	2.156–12.383	<0.001
*n*. persons/bathroom						
>1	9	62		1		
1–2	44	133		2.095	1.022–4.292	0.043
>2	24	41		1.368	1.627–7.529	0.001

**Table 4:** Univariate Exact Poisson regression of secondary transmission according to preventive measures taken by health workers to prevent infection at home.

Variable	Case	No Case	Total	IRR	95% CI	*p*
Wearing a mask at home						
No	22	42	64	1		
Yes	63	199	262	0.534	0.329–0.869	0.011
Wearing Gloves						
No	61	134	195	1		
Yes	24	107	131	0.607	0.362–0.988	0.044
Hand washing						
No	26	55	81	1	-	-
Yes	59	186	245	0.616	0.382–1.018	0.059
Wash clothes separately						
No	33	74	107	1		
Yes	52	167	219	0.650	0.413–1.039	0.0723
Individual or disposable tableware						
No	29	47	76	1	-	-
Yes	56	194	250	0.456	0.286–0.740	0.002
Cleaning surfaces with disinfectant						
No	33	59	92	1	-	-
Yes	52	182	234	0.487	0.315–0.753	0.001
Individual Bedroom						
No	19	34	53	1	-	-
Yes	66	207	273	0.490	0.294–0.816	0.001
Individual Bathroom						
No	19	34	53	1	-	-
Yes	66	207	273	0.650	0.413–1.039	0.0723

**Table 5:** Multivariate Exact Poisson regressions of secondary transmission according to preventive measures taken by health workers to prevent infection at home.

Variable	IRR *	95% CI	*p*
Wearing a mask at home	0.540	0.311–0.937	0.028
Wearing Gloves at home	0.599	0.367–0.977	0.040
individual or disposable tableware	0.517	0.313–0.855	0.010
Cleaning surfaces with disinfectant	0.510	0.312–0.833	0.007
Individual Bedroom	0.453	0.268–0.764	0.003

* Each variable is adjusted by age, sex, profession, date of onset.

## Data Availability

The datasets generated for this study are unavailable due to the data protection law.

## Supplementary Materials

The following are available online at https://www.mdpi.com/article/10.3390/epidemiologia3010001/s1, Table S1: Distribution of families and secondary cases.
