# Supplementary material for: Risk of Secondary Household Transmission of COVID-19 from Health Care Workers in a Hospital in Spain
Source: Epidemiologia (Basel). 2021 Dec 27;3(1):1–10. doi: 10.3390/epidemiologia3010001 (PMC9620916; doi:10.3390/epidemiologia3010001)
Supplement: Supplementary file 1 [file epidemiologia-03-00001-s001.zip › epidemiologia-1485555-supplementary.pdf]

epidemiologia

MDPI

Article

Risk of Secondary Household Transmission of COVID-19 from Health Care Workers in a Hospital in Spain

Miren Remón-Berrade, Sara Guillen-Aguinaga, Isabel Sarrate-Adot, Maria Pilar Garcia-Garcia, Maria del Carmen Lerga-Berruezo, Laura Guillen-Aguinaga and Francisco Guillen-Grima

Table S1. Distribution of families and secondary cases.

Number of families	Accumulated % of families	Number secondary cases	Accumulated % of secondary cases
1	1.12%	8	9.41%
2	2.25%	6	16.47%
3	3.37%	4	21.18%
4	4.49%	4	25.88%
5	5.62%	4	30.59%
6	6.74%	4	35.29%
7	7.87%	4	40.00%
8	8.99%	3	43.53%
9	10.11%	3	47.06%
10	11.24%	3	50.59%
11	12.36%	3	54.12%
12	13.48%	3	57.65%
13	14.61%	2	60.00%
14	15.73%	2	62.35%
15	16.85%	2	64.71%
16	17.98%	2	67.06%
17	19.10%	2	69.41%
18	20.22%	2	71.76%
19	21.35%	2	74.12%
20	22.47%	2	76.47%
21	23.60%	2	78.82%
22	24.72%	1	80.00%
23	25.84%	1	81.18%
24	26.97%	1	82.35%
25	28.09%	1	83.53%
26	29.21%	1	84.71%
27	30.34%	1	85.88%
28	31.46%	1	87.06%
29	32.58%	1	88.24%
30	33.71%	1	89.41%
31	34.83%	1	90.59%
32	35.96%	1	91.76%
33	37.08%	1	92.94%
34	38.20%	1	94.12%
35	39.33%	1	95.29%
36	40.45%	1	96.47%
37	41.57%	1	97.65%
38	42.70%	1	98.82%
39	43.82%	1	100.00%
40	44.94%	0	100.00%
(…)	(…)	(…)	(…)
89	100.00%	0	100.00%
